# Periprosthetic Hip Joint Infection Involving Fusobacterium nucleatum With Subsequent Isolation of Acinetobacter baumannii: A Case Report

**DOI:** 10.7759/cureus.101719

**Published:** 2026-01-17

**Authors:** Stamatios A Papadakis, Stavros Lykos, Dimitrios Pallis, Panagiotis Mantzanas, Nikolaos Paraskevopoulos, Spyros Kamariotis

**Affiliations:** 1 2nd Department of Orthopaedics, KAT General Hospital of Attica, Athens, GRC; 2 Microbiology, KAT General Hospital of Attica, Athens, GRC

**Keywords:** acinetobacter baumannii, antibiotic-loaded cement spacer, debridement antibiotics and implant retention, fusobacterium nucleatum, periprosthetic joint infection, staged revision arthroplasty, total hip arthroplasty

## Abstract

Periprosthetic joint infection (PJI) is a serious complication following total hip arthroplasty and is most commonly caused by Gram-positive organisms. Infections involving anaerobic bacteria or multidrug-resistant Gram-negative pathogens are rare and particularly challenging to manage. We report a case of periprosthetic hip infection in a female patient in whom *Fusobacterium nucleatum (F. nucleatum)* was initially isolated, followed by *Acinetobacter baumannii (A. baumannii)* later in the treatment course. Initial management with debridement, antibiotics, and implant retention (DAIR) was unsuccessful, necessitating implant removal, placement, and subsequent removal of a cement spacer, as well as prolonged targeted antimicrobial therapy, including cefiderocol. Following staged revision arthroplasty, successful infection eradication was achieved with an excellent functional outcome. This report highlights the complexity of PJI management, the potential for microbiologic evolution under antimicrobial pressure, and the importance of tailored surgical and antimicrobial strategies.

## Introduction

Total hip arthroplasty is the gold standard approach for end-stage osteoarthritis. However, periprosthetic joint infection (PJI) remains a significant complication, occurring in approximately 0.5-2% of primary procedures [1,2]. Early postoperative PJIs, particularly those occurring within the first 30 days after surgery, may be initially managed with debridement, antibiotics, and implant retention (DAIR), along with exchange of modular components, depending on symptom duration, implant stability, and the characteristics of the infecting organism [3].

The majority of PJIs are caused by Gram-positive cocci, particularly *Staphylococcus aureus (S. aureus)* and coagulase-negative staphylococci [2]. Anaerobic organisms and multidrug-resistant Gram-negative bacteria represent uncommon causes of PJI but are associated with higher treatment failure rates [4]. *Fusobacterium nucleatum (F. nucleatum)*, an anaerobic Gram-negative bacillus found in the oral and gastrointestinal flora, has been infrequently reported in osteoarticular infections but demonstrates notable tissue adherence and biofilm-forming properties [5,6]. *Acinetobacter baumannii (A. baumannii)* is an uncommon but increasingly recognized pathogen in PJIs, particularly in healthcare-associated settings, due to its capacity for biofilm formation and the acquisition of multidrug resistance [7-9]. The isolation of different pathogens at separate time points during the course of a single PJI episode is rarely described and poses significant diagnostic and therapeutic challenges.

## Case presentation

**Figure 1:**
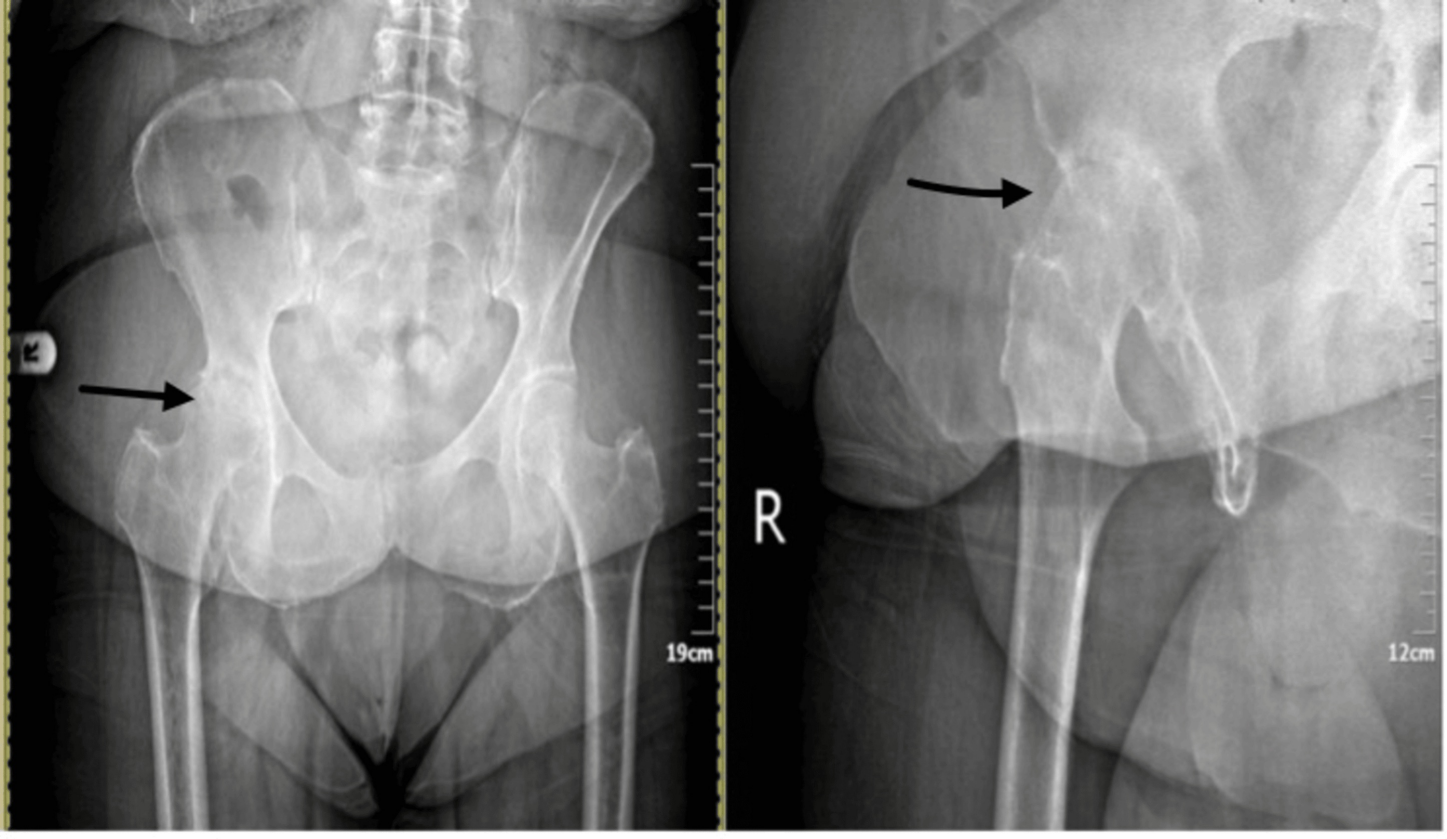
Preoperative plain radiographs

A 78-year-old female patient presented with progressive gait impairment and hip pain. Clinical evaluation revealed significant functional limitation, with a preoperative Harris Hip Score of 60. Preoperative plain radiographs demonstrated advanced degenerative changes of the hip joint consistent with end-stage osteoarthritis (Figure 1), and the patient was scheduled for primary total hip arthroplasty.

**Figure 2:**
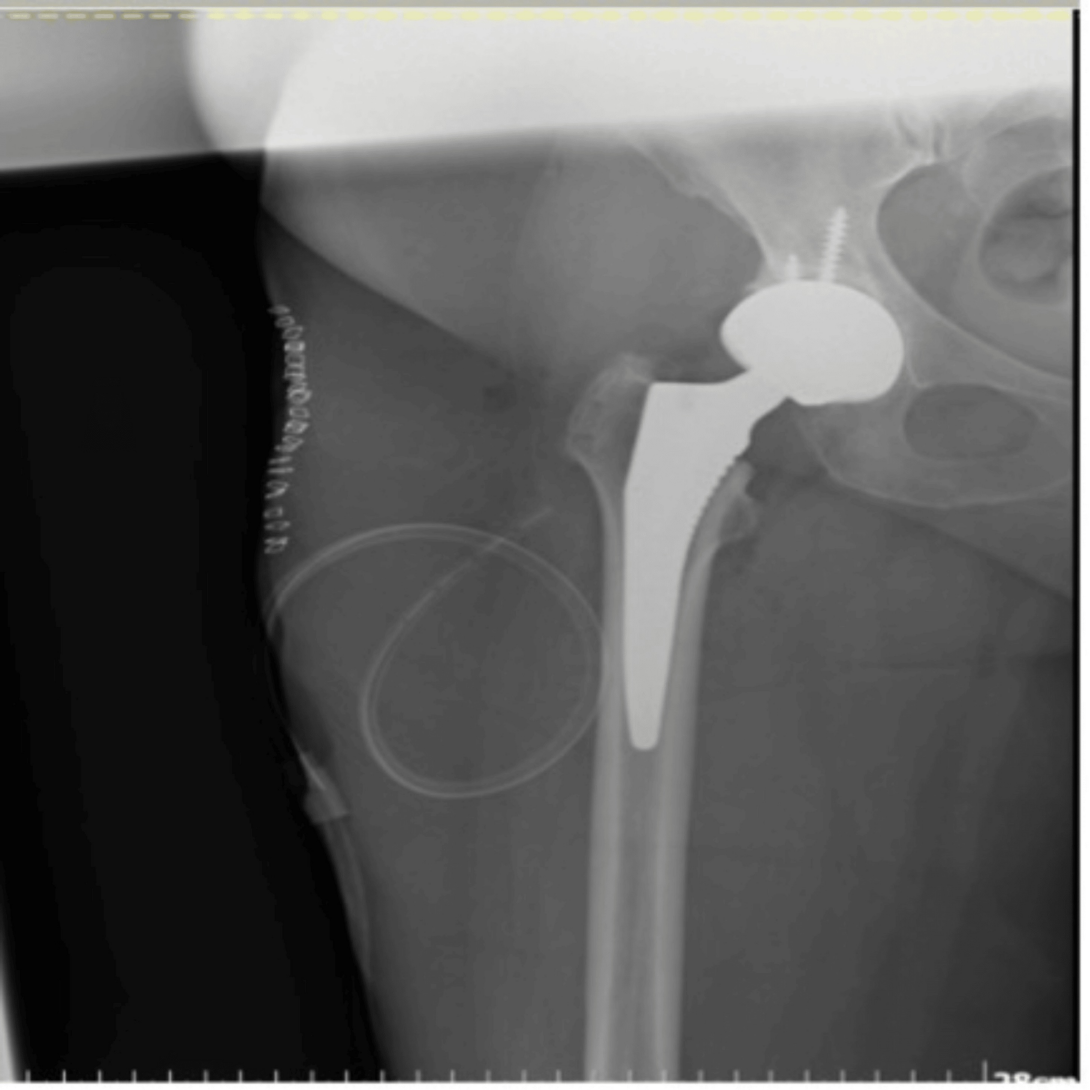
Immediate postoperative plain radiograph

**Figure 3:**
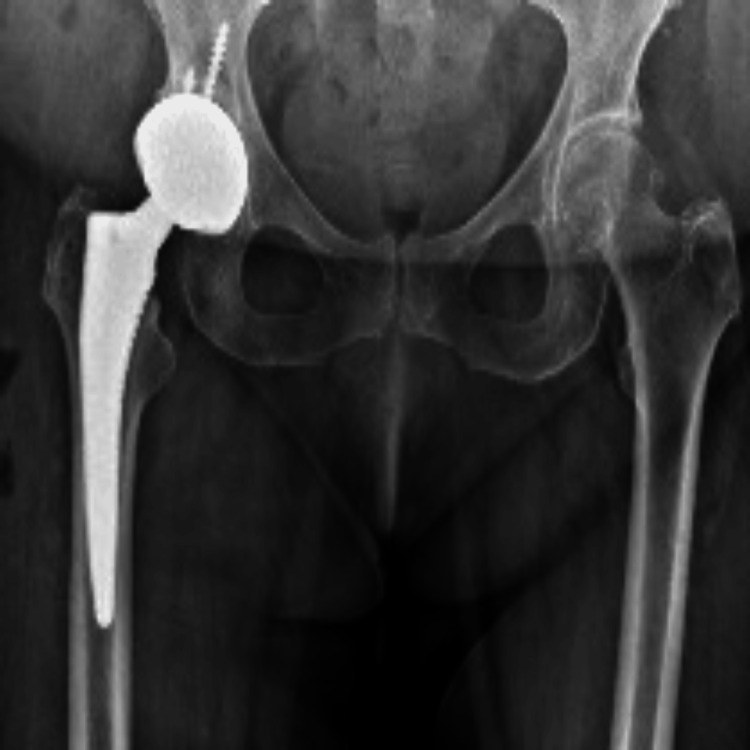
Postoperative plain radiograph

Primary total hip arthroplasty was subsequently performed. Immediate postoperative plain radiographs demonstrated satisfactory positioning of the prosthetic components without any mechanical complications (Figures 2, 3).

The initial postoperative course was uneventful. We use vancomycin with three doses post-surgery as an antibiotic protocol. Approximately 10 days postoperatively, the patient developed purulent wound drainage. A PJI was suspected, and the patient was urgently taken to the operating room. A DAIR procedure was performed, consisting of extensive irrigation and debridement with exchange of the mobile components while retaining the fixed implants. Empiric intravenous vancomycin therapy was initiated.

**Figure 4:**
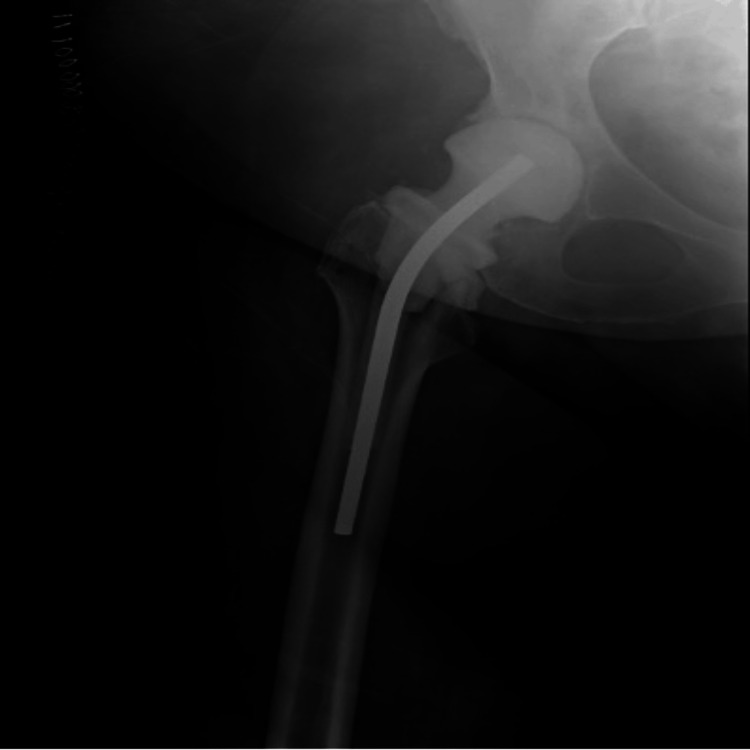
Plain radiograph after failed DAIR procedure DAIR: debridement, antibiotics, and implant retention

Microbiological analysis of intraoperative tissue samples and sonication fluid yielded growth of *F. nucleatum*. Following consultation with the infectious diseases team, meropenem was added, while vancomycin was subsequently discontinued due to a drug-related skin rash. Despite targeted antimicrobial therapy, inflammatory markers failed to normalize. Given persistent concern for infection, a subsequent surgical intervention was undertaken with complete removal of the prosthetic components and placement of an antibiotic-loaded cement spacer. Postoperative plain radiographs confirmed the presence of the spacer (Figure 4).

**Figure 5:**
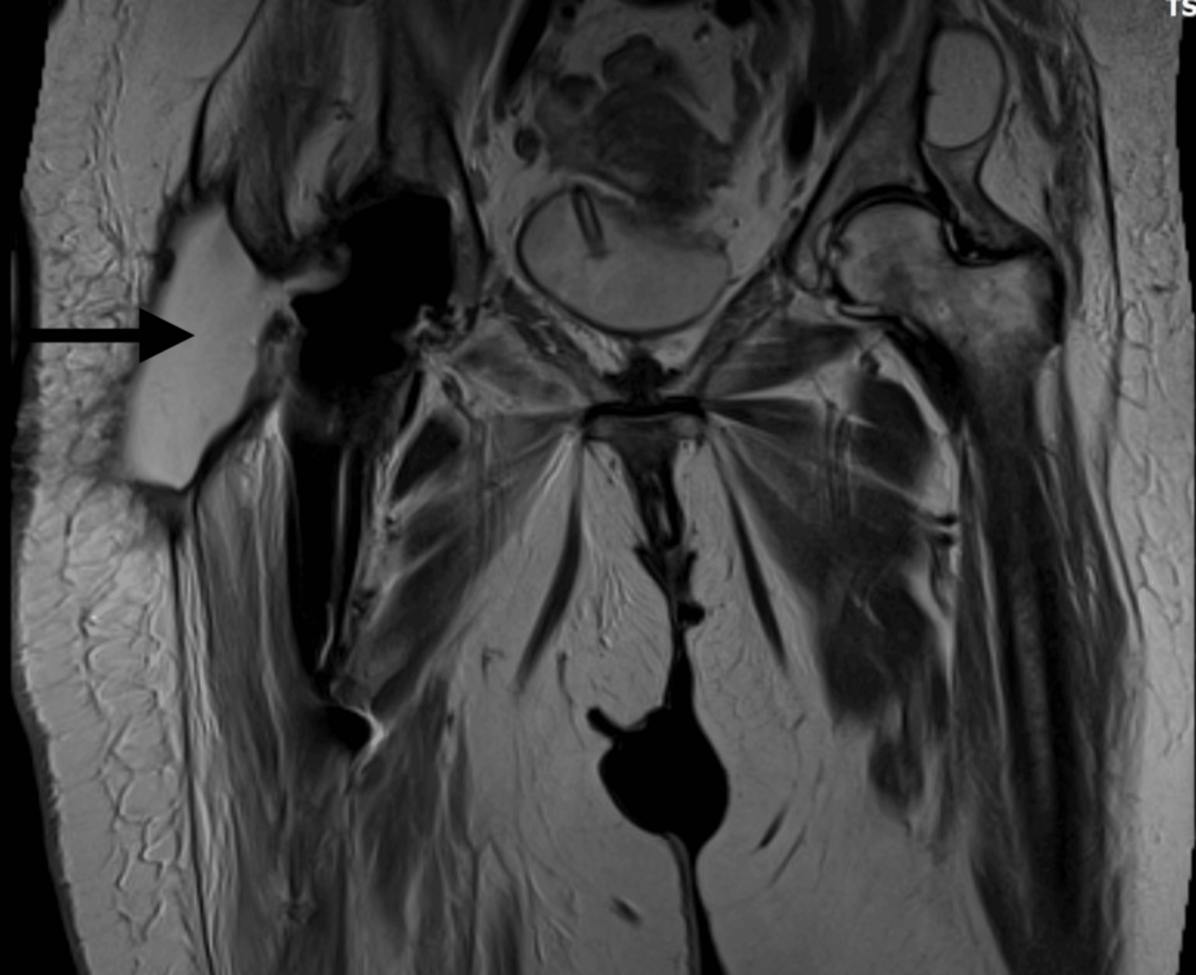
MRI demonstrating persistent periprosthetic fluid collection MRI: magnetic resonance imaging

**Figure 6:**
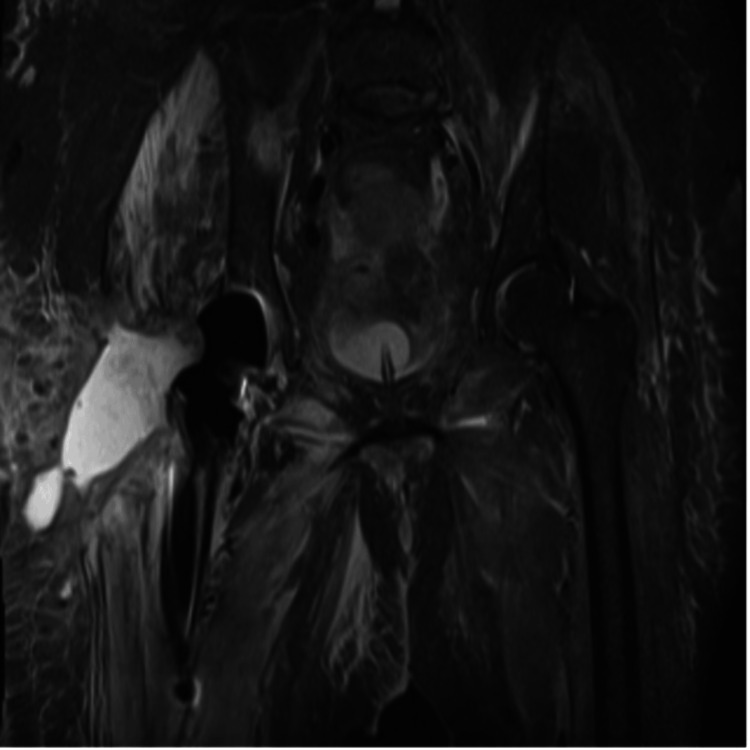
MRI during spacer phase NRI: magnetic resonance imaging

Intraoperative cultures yielded *A. baumannii *complex, exhibiting extensive drug resistance. Cefiderocol was added to the treatment. During the spacer phase, MRI demonstrated a periprosthetic fluid collection within the hip joint, consistent with ongoing inflammatory or infectious activity (Figures 5, 6).

**Figure 7:**
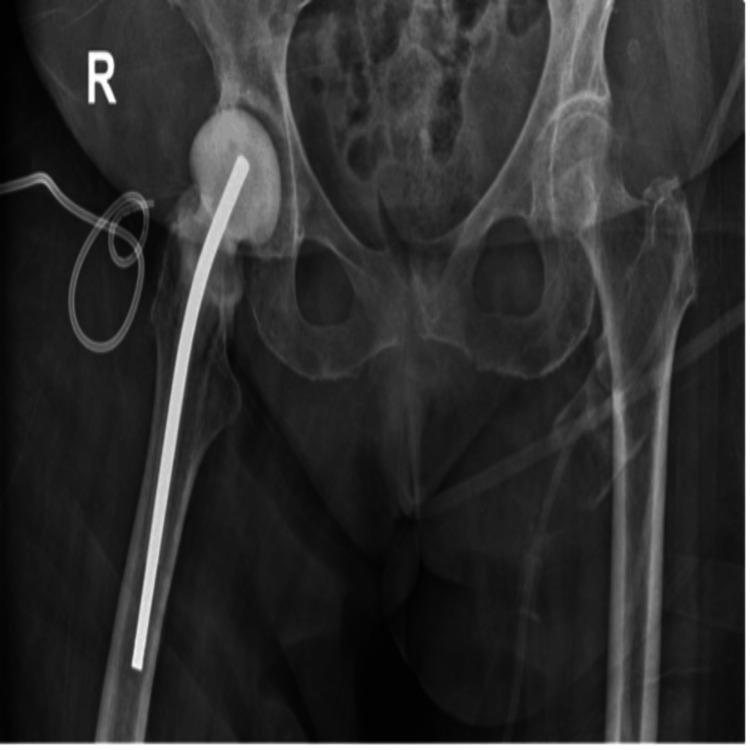
Plain radiograph during spacer interval

Plain radiographic evaluation during this period confirmed proper positioning of the cement spacer (Figure 7).

**Figure 8:**
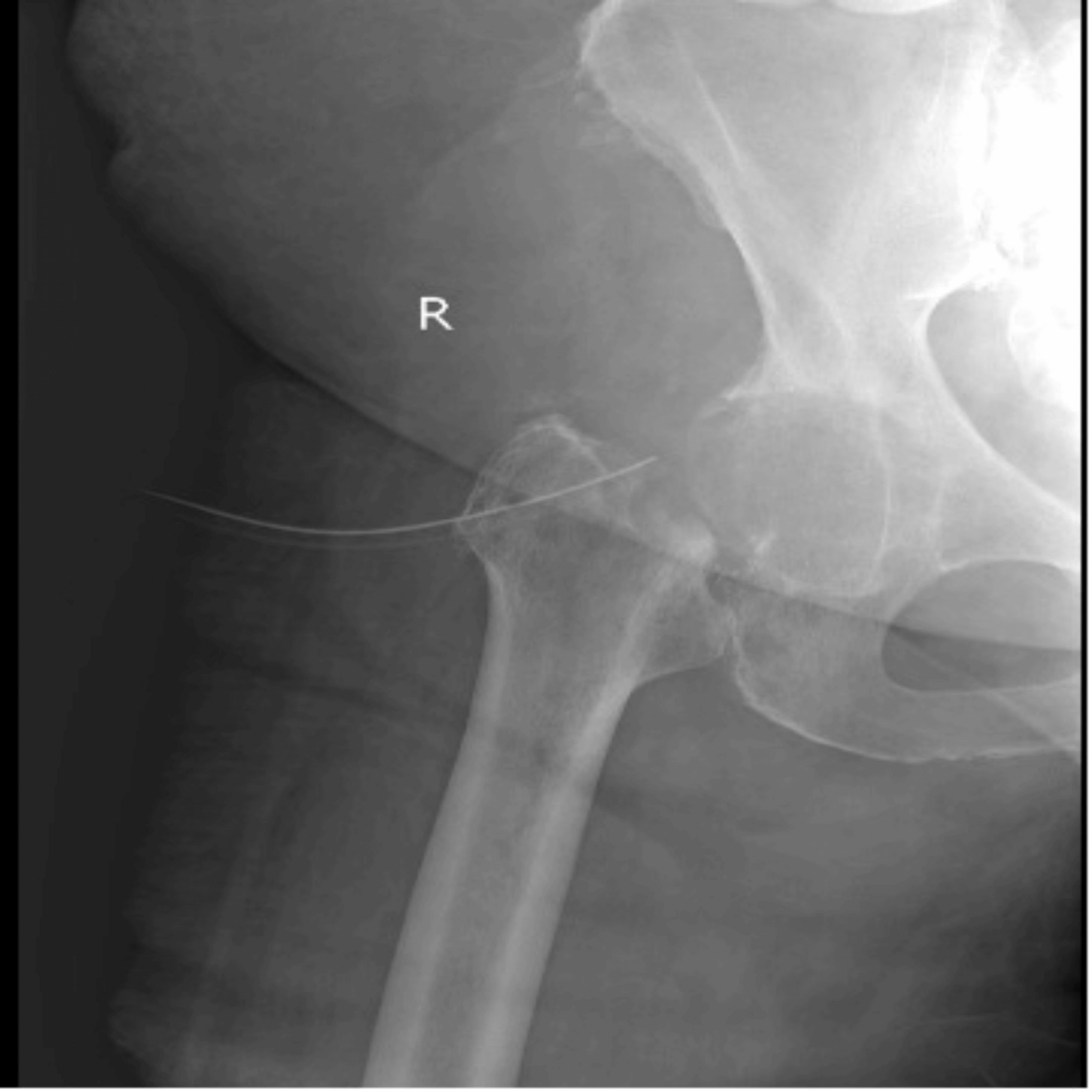
Plain radiograph after additional debridement and spacer removal

Following further surgical debridement, repeat plain radiographs demonstrated complete removal of the spacer, leaving the patient temporarily without any implant (Figure 8).

**Figure 9:**
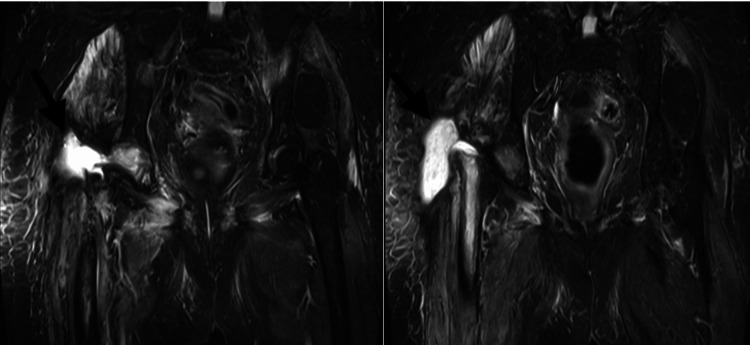
MRI showing fluid retention MRI: magnetic resonance imaging

**Figure 10:**
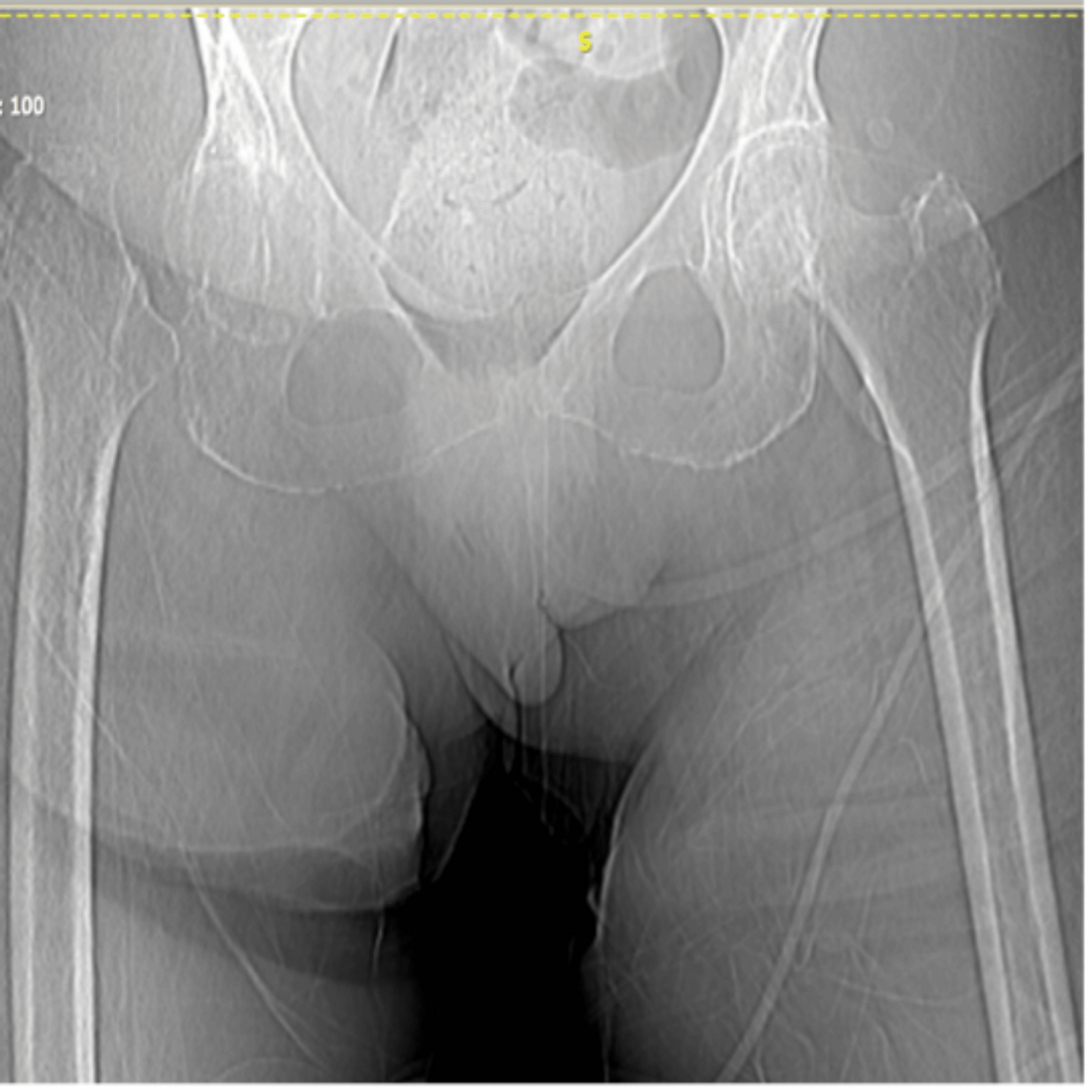
Plain radiograph during mid-term follow-up

During the spacer-free interval, MRI demonstrated persistent fluid collection within the hip joint despite the absence of any implant or spacer (Figure 9). Subsequent plain radiographic evaluation confirmed the continued absence of any implant or spacer (Figure 10).

**Figure 11:**
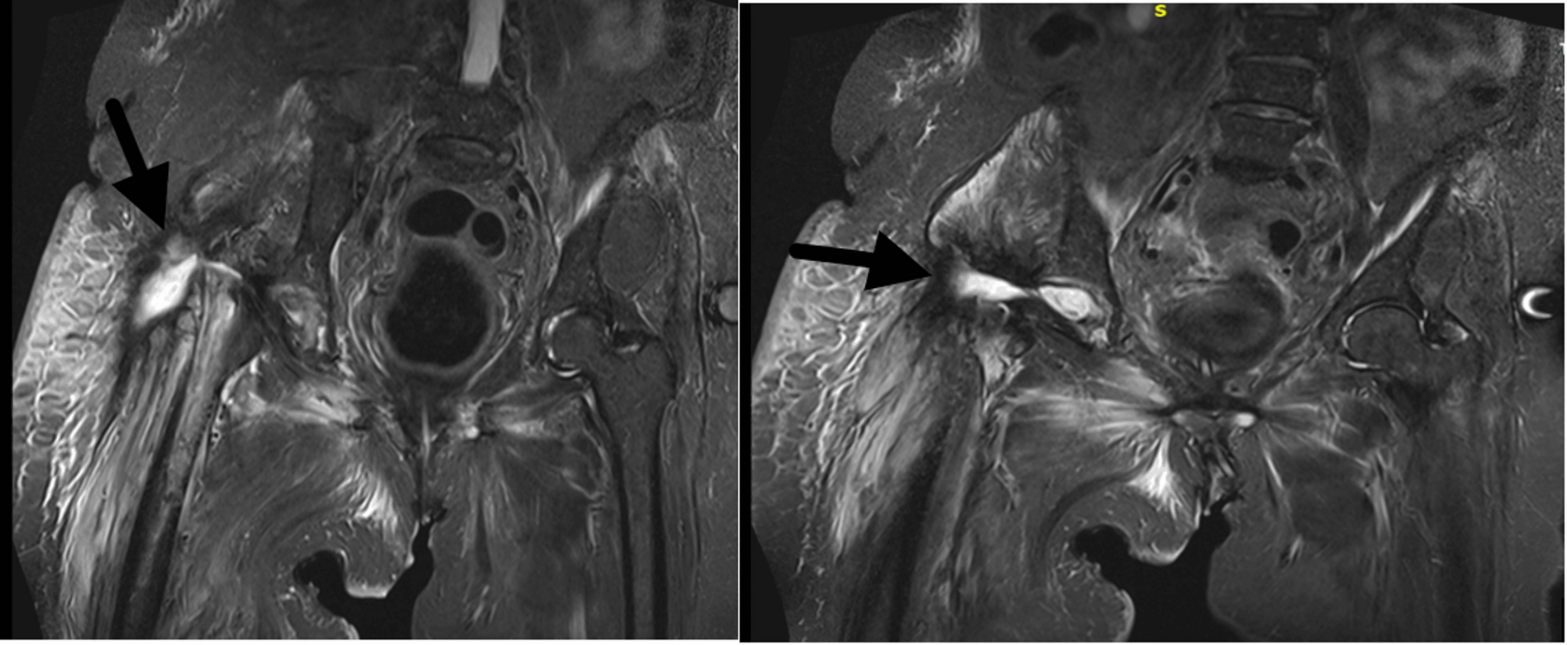
MRI demonstrating further improvement MRI: magnetic resonance imaging

**Figure 12:**
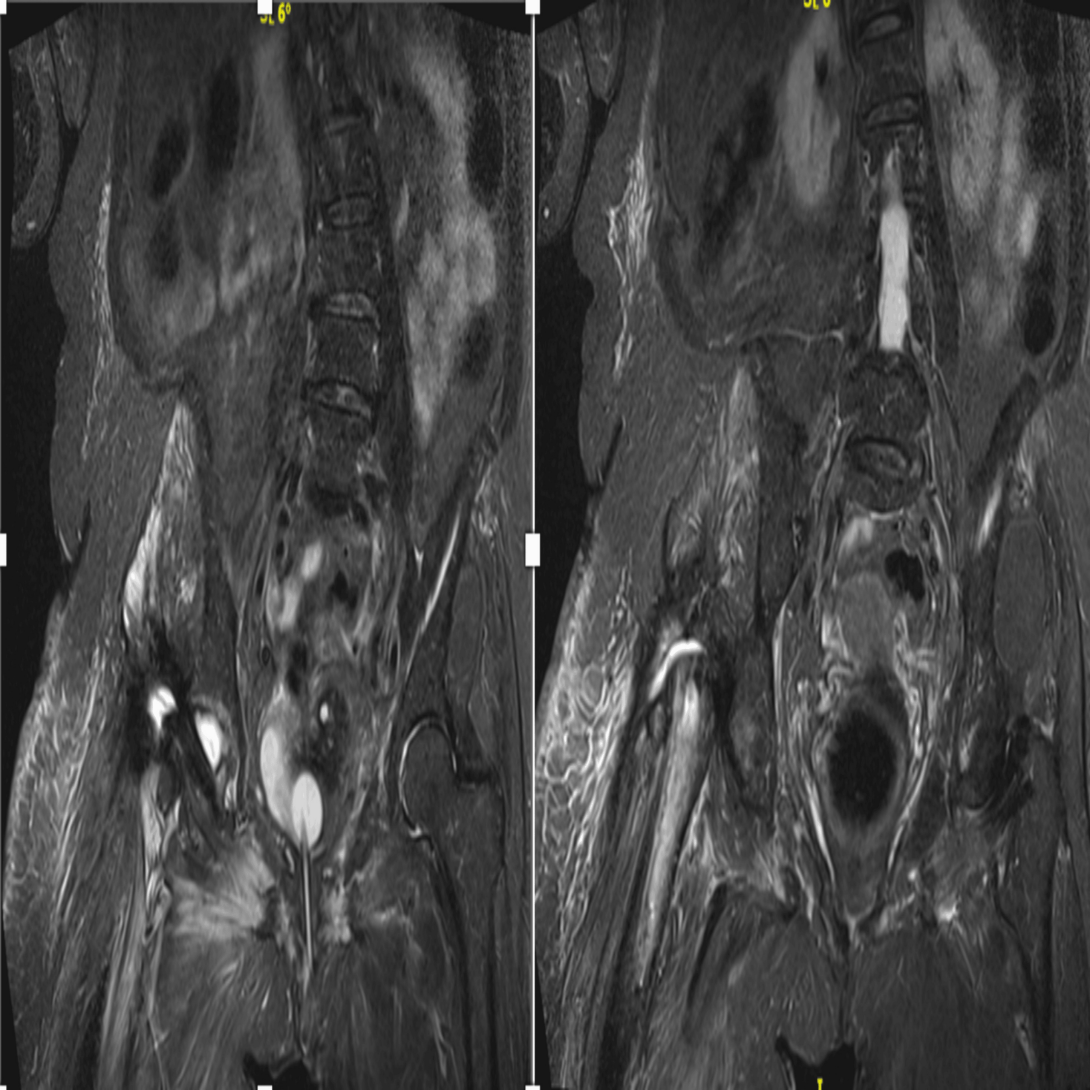
MRI prior to definitive reimplantation MRI: magnetic resonance imaging

All previous antibiotics were discontinued, and treatment with cefiderocol was initiated, followed by a prolonged course of daptomycin for two months. Follow-up MRI demonstrated minimal residual fluid collection within the hip joint, indicating interval improvement during the spacer-free period (Figures 11, 12).

**Figure 13:**
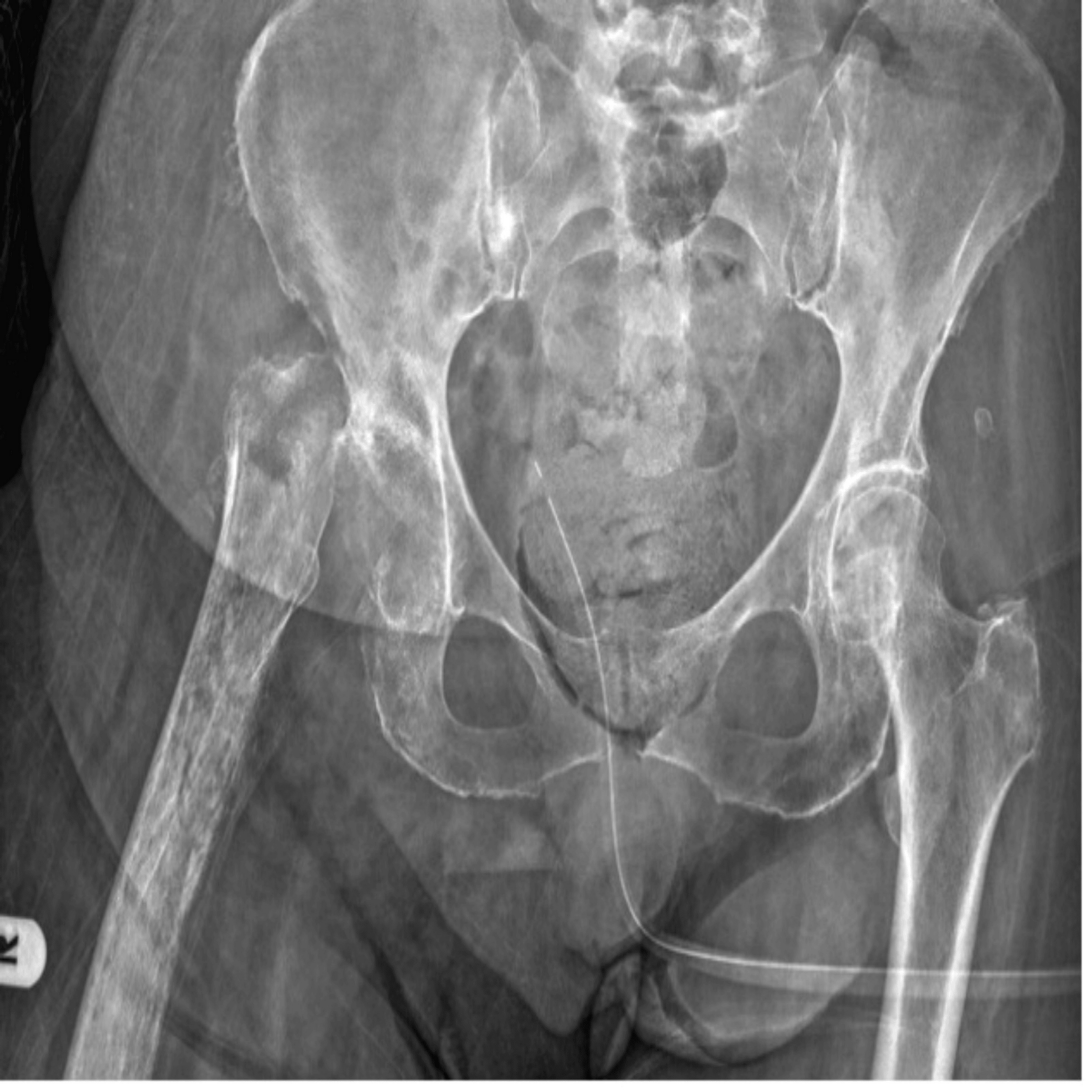
Plain radiograph before final revision surgery

Plain radiographs obtained immediately before definitive reimplantation demonstrated satisfactory bone stock and absence of acute radiographic abnormalities (Figure 13).

**Figure 14:**
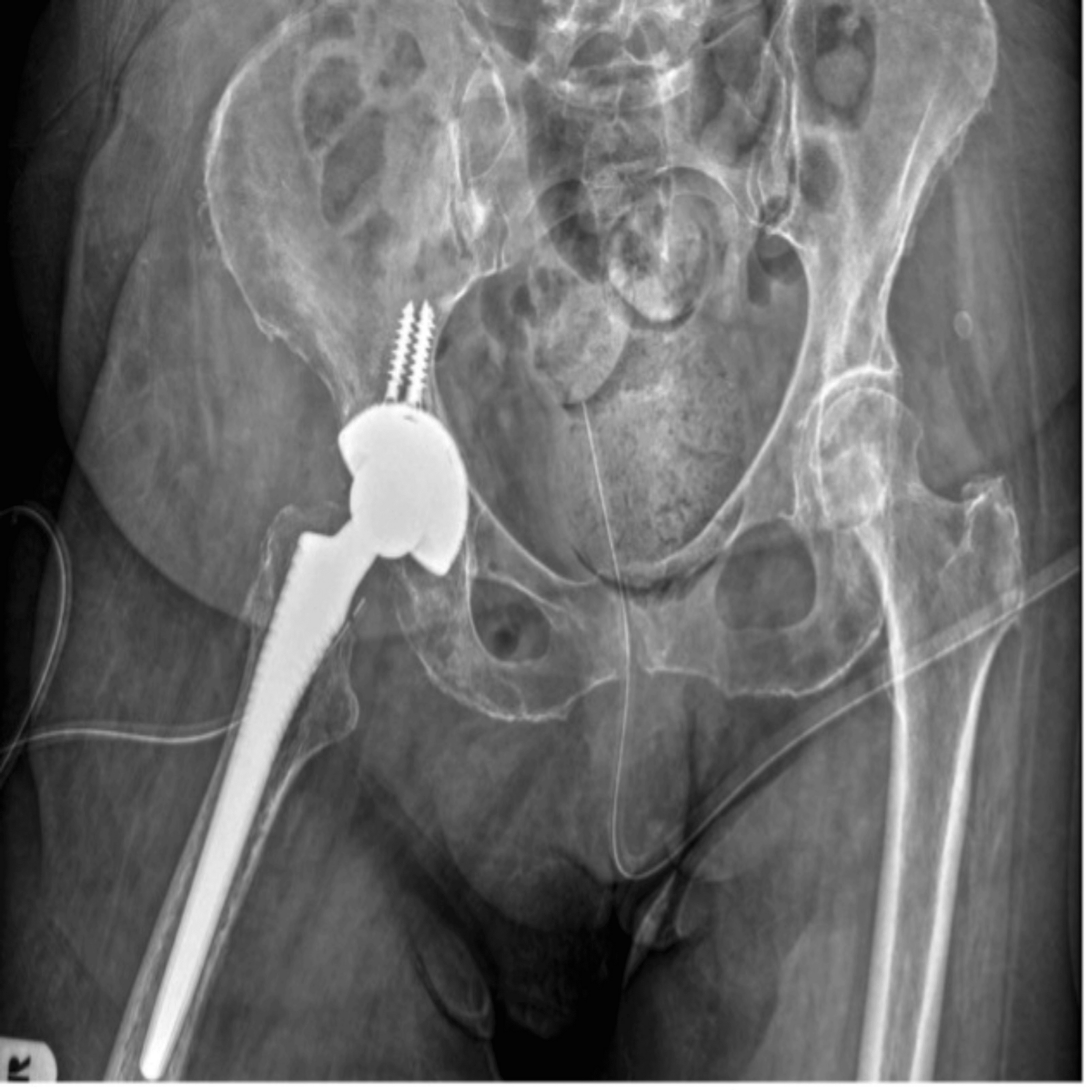
Post-reimplantation plain radiograph

The patient subsequently underwent definitive revision total hip arthroplasty. Postoperative plain radiographs confirmed proper positioning of the reimplanted prosthetic components (Figure 14).

At the six-month follow-up, the patient was pain-free, fully ambulatory, and demonstrated excellent functional recovery, with a Harris Hip Score of 90 and no clinical, laboratory, or radiological evidence of infection recurrence.

## Discussion

This report illustrates the complexity of managing PJI, particularly when rare and multidrug-resistant pathogens are involved. Although DAIR is an accepted initial strategy in select early PJIs, failure rates remain substantial, especially in infections caused by virulent or resistant organisms [10]. The initial isolation of *F. nucleatum* is notable. Although it is rarely implicated in PJIs, *F. nucleatum* demonstrates strong adhesion to host tissues and participates in the formation of polymicrobial biofilms, which may contribute to its persistence in deep-seated infections [5,6]. Anaerobic PJIs may also be underdiagnosed due to limitations in routine culture techniques [4]. The subsequent isolation of *A. baumannii* underscores the dynamic microbiology that may emerge during prolonged antimicrobial exposure. *A. baumannii* is well recognized for its ability to form robust biofilms on abiotic surfaces, persist in the hospital environment, and rapidly develop multidrug resistance [7-9,11]. These characteristics significantly impair antimicrobial efficacy and are associated with higher rates of failure of implant-retention strategies [8,12].

In the present case, repeated surgical debridements, including a period of resection arthroplasty without a spacer, were intentionally undertaken to achieve adequate source control and reduce the bacterial burden. Definitive reimplantation was deliberately delayed until inflammatory markers had normalized and imaging demonstrated minimal residual fluid collection, reflecting a clinical assessment of a reduced risk for persistent infection [10,12,13]. Failure of DAIR ultimately necessitated escalation of care to implant removal and staged revision arthroplasty, which remains the gold standard for persistent or complex PJIs [14]. Cefiderocol, a siderophore cephalosporin with activity against multidrug-resistant Gram-negative pathogens, including *A. baumannii*, was used as rescue therapy when standard antimicrobial options were exhausted, consistent with emerging evidence in the treatment of complex bone and joint infections [15].

## Conclusions

PJI may involve uncommon pathogens with evolving microbiology during the treatment course. Infections caused by anaerobic and multidrug-resistant Gram-negative organisms require heightened vigilance, aggressive surgical source control, and individualized antimicrobial strategies. Timely escalation to staged revision arthroplasty and careful timing of reimplantation can lead to durable infection eradication and excellent functional outcomes.

## References

[1] Tande AJ, Patel R (2014). Prosthetic joint infection. Clin Microbiol Rev.

[2] Kapadia BH, Berg RA, Daley JA, Fritz J, Bhave A, Mont MA (2016). Periprosthetic joint infection. Lancet.

[3] Osmon DR, Berbari EF, Berendt AR (2013). Diagnosis and management of prosthetic joint infection: clinical practice guidelines by the Infectious Diseases Society of America. Clin Infect Dis.

[4] Hsieh PH, Lee MS, Hsu KY, Chang YH, Shih HN, Ueng SW (2009). Gram-negative prosthetic joint infections: risk factors and outcome of treatment. Clin Infect Dis.

[5] Brook I (2002). Anaerobic infections in orthopedic surgery. Clin Orthop Relat Res.

[6] Han YW (2015). Fusobacterium nucleatum: a commensal-turned pathogen. Curr Opin Microbiol.

[7] Peleg AY, Seifert H, Paterson DL (2008). Acinetobacter baumannii: emergence of a successful pathogen. Clin Microbiol Rev.

[8] Garnacho-Montero J, Timsit JF (2019). Managing Acinetobacter baumannii infections. Curr Opin Infect Dis.

[9] Longo F, Vuotto C, Donelli G (2014). Biofilm formation in Acinetobacter baumannii. New Microbiol.

[10] Lora-Tamayo J, Murillo O, Iribarren JA (2013). Outcome of debridement, antibiotics, and implant retention in early prosthetic joint infection. Clin Infect Dis.

[11] Davis KA, Moran KA, McAllister CK, Gray PJ (2005). Multidrug-resistant Acinetobacter extremity infections in soldiers. Emerg Infect Dis.

[12] Rodríguez-Pardo D, Pigrau C, Lora-Tamayo J (2014). Gram-negative prosthetic joint infection: outcome of a debridement, antibiotics and implant retention approach. A large multicentre study. Clin Microbiol Infect.

[13] Shukla SK, Ward JP, Jacofsky MC, Sporer SM, Paprosky WG, Della Valle CJ (2010). Perioperative testing for persistent sepsis following resection arthroplasty of the hip for periprosthetic infection. J Arthroplasty.

[14] Zimmerli W, Trampuz A, Ochsner PE (2004). Prosthetic-joint infections. N Engl J Med.

[15] Falcone M, Tiseo G, Nicastro M (2021). Cefiderocol as rescue therapy for Acinetobacter baumannii and other carbapenem-resistant gram-negative infections in intensive care unit patients. Clin Infect Dis.

